# Interplay between periodic stimulation and GABAergic inhibition in striatal network oscillations

**DOI:** 10.1371/journal.pone.0175135

**Published:** 2017-04-06

**Authors:** Jovana J. Belić, Arvind Kumar, Jeanette Hellgren Kotaleski

**Affiliations:** 1 Science for Life Laboratory, School of Computer Science and Communication, KTH Royal Institute of Technology, Stockholm, Sweden; 2 Department of Computational Science and Technology, School of Computer Science and Communication, KTH Royal Institute of Technology, Stockholm, Sweden; 3 Bernstein Center Freiburg, University of Freiburg, Freiburg, Germany; 4 Department of Neuroscience, Karolinska Institute, Stockholm, Sweden; University Paris 6, FRANCE

## Abstract

Network oscillations are ubiquitous across many brain regions. In the basal ganglia, oscillations are also present at many levels and a wide range of characteristic frequencies have been reported to occur during both health and disease. The striatum, the main input nucleus of the basal ganglia, receives massive glutamatergic inputs from the cortex and is highly susceptible to external oscillations. However, there is limited knowledge about the exact nature of this routing process and therefore, it is of key importance to understand how time-dependent, external stimuli propagate through the striatal circuitry. Using a network model of the striatum and corticostriatal projections, we try to elucidate the importance of specific GABAergic neurons and their interactions in shaping striatal oscillatory activity. Here, we propose that fast-spiking interneurons can perform an important role in transferring cortical oscillations to the striatum especially to those medium spiny neurons that are not directly driven by the cortical oscillations. We show how the activity levels of different populations, the strengths of different inhibitory synapses, degree of outgoing projections of striatal cells, ongoing activity and synchronicity of inputs can influence network activity. These results suggest that the propagation of oscillatory inputs into the medium spiny neuron population is most efficient, if conveyed via the fast-spiking interneurons. Therefore, pharmaceuticals that target fast-spiking interneurons may provide a novel treatment for regaining the spectral characteristics of striatal activity that correspond to the healthy state.

## Introduction

The basal ganglia (BG) comprise the largest subcortical system in the brain and play a crucial role in motor control and cognitive processes [1–8]. Dysfunction of neural pathways between cortex and the BG circuitry is linked to many brain disorders, such as Parkinson’s disease (PD), L-DOPA-induced dyskinesia, Tourette’s syndrome, impulse control disorders, pathological gambling and Huntington’s disease [9–17]. The striatum is the largest and main input nucleus of the BG, receiving glutamatergic inputs from all cortical areas and thalamus [18–22]. About 95% of neurons in the striatum are medium spiny neurons (MSNs) that form the only output from the striatum [23–24]. Two of the most examined sources of GABAergic inhibition into MSNs are the feedback inhibition (FB) from the axon collaterals of the MSNs themselves, and the feedforward inhibition (FF) via the small population (1–2% of striatal neurons) of fast-spiking interneurons (FSIs) [25–28]. While feedback inhibition is made up of many weak inputs the feedforward inhibition is powerful, and spiking in a single FSI is capable of significantly delaying spike generation in a large number of postsynaptic medium spiny neurons [29–34]. High firing rate and uncorrelated spiking of FSIs may further amplify the effect of feedforward inhibition on the MSNs [35–36].

Recently, oscillations (20–80 Hz) have been observed at the level of individual MSNs firing and local field potential in the striatum of both awake and anaesthetized animals [17, 37–39]. The mechanisms underlying the emergence of these oscillations remain unclear. Purely inhibitory network such as the striatum can exhibit network oscillations but this requires very strong input excitation combined with strong recurrent inhibition [40]. However, given the weak and sparse recurrent inhibition between any two MSNs [41] it seems unlikely that the experimentally observed oscillations are locally generated by the inhibitory network of the striatum, although collective FB inhibition from thousands of MSNs due to their large number can generate substantial inhibition [42]. A second possibility is that striatal neurons have sub-threshold resonance properties and when the network is driven strongly enough the oscillations become apparent, but it has been found that MSNs do not show any resonance behavior [43]. A third possibility is that the experimentally observed oscillations in the striatum are in fact cortical oscillations transmitted by the cortico-striatal projections. While the transmission of firing rates and correlations via the cortico-striatal projections have been investigated previously [44–45] it is not clear to what extent cortico-striatal projections can transfer oscillations over a wide range of frequencies and what roles do the FB and FF inhibitions play in this process.

Here, we use a spiking neuron network model of the striatum to isolate the mechanisms underlying the transmission of cortical oscillations to not only the MSNs that receive direct cortical inputs but also to the other unstimulated MSNs. We systematically studied the effect of FB and FF inhibitions, the density of outgoing projections and the overall activity of striatal cells on the oscillatory activity of the striatum. We show that FSIs, despite their asynchronous spiking, play a crucial role in efficient propagation of cortical oscillations to the MSNs, especially the ones that did not receive direct cortical oscillations. In the absence of FSI inputs, spread of oscillations to the unstimulated MSNs requires either unphysiologically high baseline firing rate of the MSNs or direct cortical stimulation of a very large fraction of MSNs. Our findings reveal a new role of FSIs in modulating the transfer of information from the cortex to striatum and by modulating the activity and properties of the FSIs, striatal oscillations can be controlled very efficiently.

## Materials and methods

### Neuron models

The neurons in the network were modeled as leaky integrate-and-fire neurons. We considered two types of striatal neurons: medium spiny neurons and fast-spiking neurons. Subthreshold dynamics of the membrane potential $V_{i}^{MSN}\left(t\right)$ of a MSN *i* was described by the following equation:

$$C^{MSN}\frac{d}{dt}V_{i}^{MSN}\left(t\right)+G_{rest}^{MSN}\left[V_{i}^{MSN}\left(t\right)-V_{rest}^{MSN}\right]=I_{i}^{MSN}\left(t\right)$$ (1)

where $I_{i}^{MSN}\left(t\right)$ was the total synaptic input current to the neuron, and *C*^*MSN*^ and $G_{rest}^{MSN}$ denoted the passive electrical cell properties, the capacitance and conductance of its membrane at rest ($V_{rest}^{MSN}$), respectively. When the membrane potential reached a fixed spiking threshold $\;V_{th}^{MSN}$, a spike was emitted, and the membrane potential was reset to the resting value.

The subthreshold dynamics of the membrane potential $V_{i}^{FSI}\left(t\right)$ of a FSI *i* was described similarly by the following equation:

$$C^{FSI}\frac{d}{dt}V_{i}^{FSI}\left(t\right)+G_{rest}^{FSI}\left[V_{i}^{FSI}\left(t\right)-V_{rest}^{FSI}\right]=I_{i}^{FSI}\left(t\right)$$ (2)

The initial membrane potentials of both MSNs and FSIs were chosen from a uniform distribution from -86.3 to -55 mV and from -82 mV to -65 mV, respectively, in order to avoid any unwanted synchrony, caused by the initial conditions in the simulation runs.

We have chosen to use the simple leaky integrate-and-fire neuron model that lacks oscillatory features and, therefore, all frequency responses are a direct outcome of network interactions [46]. More complex neuron models, such as resonate-and-fire [47] and properly parameterized Hodgkin-Huxley [48] models have oscillatory sub threshold dynamics and using such neuron models would have made it difficult to determine the role of striatal network interactions in the transfer of cortical oscillations to the MSNs.

### Network

**Fig 1:**
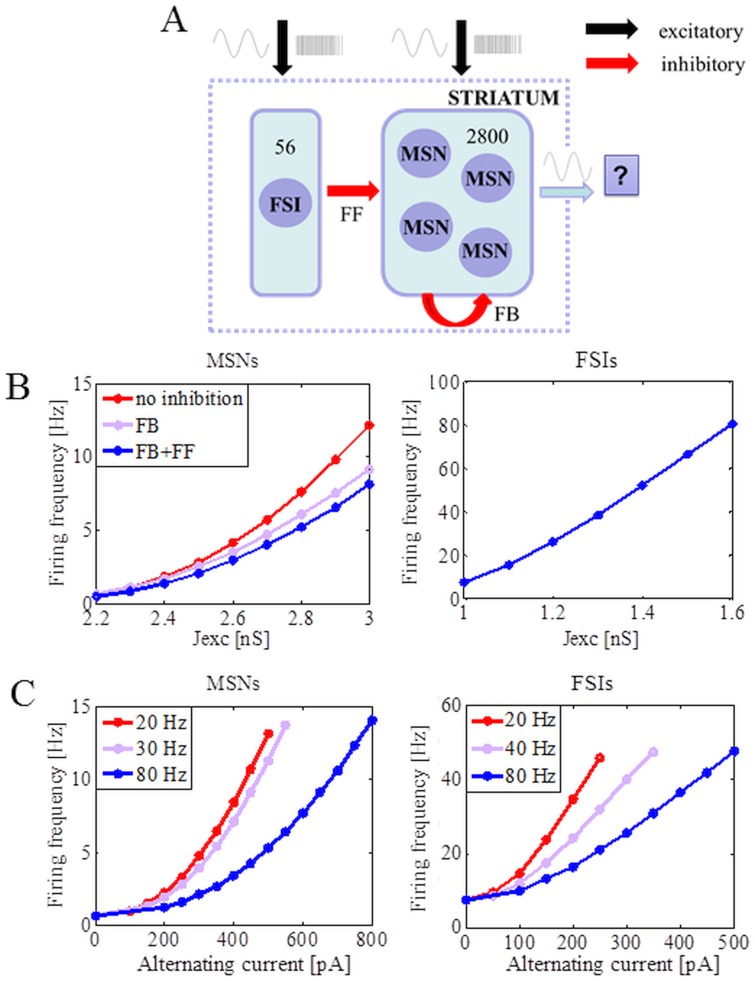
Input-output transfer functions of MSNs and FSIs in a network of striatum. (A) Schematic of the network model of striatum with MSNs receiving inhibitory inputs from FSIs and other MSNs. The selected populations, which depend on the particular studied setup, of FSIs and MSNs receive sinusoidal current and background activity. The rest of the neurons (that do not receive oscillatory inputs) receive only excitatory and uncorrelated Poisson synaptic inputs to achieve the different realistic firing rates of MSNs and FSIs. (B) Change in the firing rate of MSNs as a function of the strength of excitatory inputs (left panel). The neurons were driven by Poisson type spiking inputs (600 Hz). The effect of feedforward and feedback inhibition in reducing the MSNs firing rate increased with an increase in the firing rate of MSNs. Change in the firing rate of FSIs as a function of the strength of excitatory input when FSIs were driven by Poisson type spiking input (600 Hz; right panel). (C) Effect of the amplitude and frequency of sinusoidal current on the output firing rate of MSNs (left panel) and FSIs (right panel). The MSNs and FSIs received sinusoidal current inputs at different amplitudes and frequencies in addition to a fixed Poisson type spiking inputs that set the baseline firing rate of the MSNs (<1 Hz) and FSIs (<10 Hz).

It has been estimated that there are around 2800 MSNs located within the volume of the dendrites of one medium spiny cell. MSNs are the dominant neuron type in the striatum (up to 95% in rodents [49]), and the radius of their axonal and dendritic arborisations are both around 200 μm. There are at least two main inhibitory circuits in the striatum that are activated by cortical inputs and that control firing in MSNs. The first is FF inhibition via the small population of FSIs, and the second is FB inhibition from the axon collaterals of the MSNs themselves. Therefore, we simulated a network of two types of GABAergic neurons, 2800 MSNs and 56 FSIs, which was driven with external inputs (see section 1.4) corresponding to an external stimulation from the cortex. A scheme of the striatal network model is shown in Fig 1A. The connection probability between the MSNs was equal to 0.18 [50]. Each MSN received inhibitory inputs from on average 11 FSIs, resulting in 20% connectivity from FSIs to MSNs [30].

### Synapses

Excitatory synaptic input was modeled by transient conductance changes using the alpha-function such that:

$$g_{exc}^{\mu }=\{\begin{array}{ll} J_{exc}^{\mu }\frac{t}{\tau exc}e^{1-\frac{t}{\tau exc}} & for\;t\geq 0\\ 0 & for\;t\;\leq 0 \end{array},$$ (3)

where μ ϵ {MSN, FSI}, *τ*_*exc*_ and $J_{exc}^{\mu }$ denoted the rise times for the excitatory synaptic inputs and the peak amplitude of the conductance transient (‘strength’ of the synapses), respectively (Fig 1B). Inhibitory synaptic input was modeled similarly:

$$g_{inh}^{ɤ}=\{\begin{array}{ll} J_{inh}^{ɤ}\frac{t}{\tau inh}e^{1-\frac{t}{\tau inh}} & for\;t\geq 0\\ 0 & for\;t\;\leq 0 \end{array},$$ (4)

where ɤ ϵ {FF, FB}. The total excitatory conductance $G_{exc,i}^{MSN}\left(t\right)$ in a MSN *i* was equal to:

$$G_{exc,i}^{MSN}\left(t\right)=\Sigma_{m\epsilon K_{i}^{MSN}}\Sigma_{n}g_{exc}^{MSN}\left(t-t_{mn}^{CTX}\right).$$ (5)

Here, $K_{i}^{MSN}$ denoted the set of excitatory synapses projecting onto MSN *i*. The inner sum runs over the sequence of spikes (*n*’s), and the set $t_{mn}^{CTX}$ represented the spike times of the excitatory neuron *m*. The total inhibitory conductance $G_{inh,i}^{MSN}\left(t\right)$ in a MSN *i* was given by:

$$G_{inh,i}^{MSN}\left(t\right)=\Sigma_{l\epsilon K_{i}^{FF}}\Sigma_{k}g_{inh}^{FF}\left(t-t_{lk}^{FF}-D^{FF}\right)+\Sigma_{l\prime \epsilon K_{i}^{FB}}\Sigma_{k\prime }g_{inh}^{FB}\left(t-t_{l^{\prime }k^{\prime }}^{FB}-D^{FB}\right).$$ (6)

MSNs mainly project onto the dendrites of other MSNs and FSIs tend to project onto the soma, therefore we used a shorter delay for FF inhibition (*D*^*FF*^ = 1 *ms*) compared to the delay used for FB inhibition (*D*^*FB*^ = 2 *ms*). Similarly, $K_{i}^{FF}$ and $K_{i}^{FB}$ were the sets of presynaptic FSIs and MSNs projecting to MSN *i*. The excitatory conductance $G_{exc}^{FSI}\left(t\right)$ in a FSI *i* was given by:

$$G_{exc,i}^{FSI}\left(t\right)=\Sigma_{m\prime \epsilon K_{i}^{FSI}}\Sigma_{n\prime }g_{exc}^{FSI}\left(t-t_{m\prime n\prime }^{CTX}\right).$$ (7)

The total synaptic current onto a MSN *i* was:

$$I_{i}^{MSN}\left(t\right)=-G_{exc,i}^{MSN}\left(t\right)\left[V_{i}^{MSN}\left(t\right)-V_{exc}^{MSN}\right]-G_{inh,i}^{MSN}\left(t\right)\left[V_{i}^{MSN}\left(t\right)-V_{inh}^{MSN}\right],$$ (8)

and the total synaptic current onto a FSI i was:

$$I_{i}^{FSI}\left(t\right)=-G_{exc,i}^{FSI}\left(t\right)\left[V_{i}^{FSI}\left(t\right)-V_{exc}^{FSI}\right].$$ (9)

$V_{exc}^{MSN}$ and $V_{inh}^{MSN}$ were the reversal potentials of the excitatory and inhibitory synaptic currents of MSNs, respectively, and similarly $V_{exc}^{FSI}$ denoted the reversal potentials of the excitatory currents of FSIs.

Based on experimental results, the corresponding strengths of the inhibitory synapses for FF and FB inhibitions in our model, $J_{inh}^{FF}$ and $J_{inh}^{FB}$, were set to 3 nS and 0.5 nS, respectively, although we varied the strength of the connections over a range around the experimental values to investigate their influence [30,51]. The parameter values for both MSNs and FSIs in our network model are summarized in Table 1.

**Table 1:** Parameter values of the model neurons used in this study.

Quantity	MSN	FSI
C [pF]	120 [33]	100 [33]
V_rest_ [mV]	-86.3 [51]	-82 [33, 52]
V_t_ [mV]	-43.75 [51]	-55 [53]
V^μ^_exc_ [mV]	0	0
V^μ^_inh_ [mV]	-65 [49, 54]	-75 [53]
τ_exc_ [ms]	2.	2.
τ_inh_ [ms]	0.3	0.3
G_rest_ [nS]	15.175 [51]	10 [54]

### External input

The selected population (FSIs, MSNs or both) and number (all or only the portion) of neurons, which depended on the particular studied setup, received the background activity and sinusoidal current, corresponding to an external stimulation from other brain areas, given for neuron *i* as:

$$I_{i,\;ext}^{\mu }\left(t\right)=A_{i}^{\mu }\text{sin}\left(C_{f}t+\delta_{i}\right),$$ (10)

where μ ϵ {MSN, FSI}. The amplitude of oscillations $A_{i}^{\mu }$ depended on the type of neuron and the driving frequency *C*_*f*_. It was always set in the 0.9 $A_{max}^{\mu }-A_{max}^{\mu }$ range so that the randomness could be imposed (Fig 1C). The sinusoidal input was present from the start of the simulation, and δ was a random starting phase in the 0°– 180° range. The rest of the population (that did not receive oscillatory inputs) received external, excitatory and uncorrelated Poisson synaptic inputs set to 600 Hz to achieve the different realistic firing rates of MSNs and FSIs (Fig 1B). The background activity constrained MSNs to fire < 1 Hz and FSIs < 10 Hz.

### Network simulation

All network simulations were written in the Python interface to NEST [55]. The dynamical equations were integrated at a fixed temporal resolution of 0.01 *ms* using the fourth order Runge-Kutta method. This small time step was necessary because of the fast dynamics of the FSIs. Stimulation ran for 1 *s* and each explored scenario was repeated and averaged 10 times, yielding 10 trials.

### Model limitations

In this study, we wanted to better understand the sole striatal network response to oscillatory input and therefore we reduced our model in order to tackle this complex question. The 2800 MSNs located within the volume of the dendrites of one medium spiny cell constitute only a small volume of striatum and, hence, it is reasonable to assume a distance-independent random connectivity in the simulated subpart of the network. It is worth mentioning that our results remained qualitatively unchanged when we assumed distance-dependent connectivity (data not shown) or included GABAergic synapses between FSIs. In the striatum, FSIs are also interconnected by gap junctions, but it has been predicted that the synchronization effects due to gap junctions are quite low when they fire at moderate intensity [56]. Modulatory effects of tonically active and dopaminergic neurons were taken into account by changing the effective strength of the FB and FF inhibitions based on experimental data. Persistent low-threshold spiking (PLTS) neurons are also known to inhibit MSNs, but their output is relatively weak and sparse, therefore these neurons were not included in this model framework [33].

### Data analysis

Network activity in the population that consisted of MSNs was evaluated by counting the overall spiking activity in time bins of 5 ms, which was equal to the number of active cells per time bin. To estimate the strength of oscillations we used the fact that oscillations introduce peaks in the power spectral density of the population activity. Therefore, we estimated the spectrum *S*(*f*) of the population activity, directly from the spike trains, using the Fast-Fourier-Transform with the sampling frequency set to 200 Hz. We defined the oscillations index as the relative power in the frequency band of interest as (±5 Hz around the driving frequency of interest *C*_*f*_):

$$OI\left[pop\right]=\frac{\int_{C_{f}-5}^{C_{f}+5}S\left(f\right)df}{\int_{1}^{Fs/2}S\left(f\right)df}.$$ (11)

When the network was oscillating strongly, most of the power was contained in the *C*_*f*_ ± 5 band and, as a result, *OI* values were higher.

To measure the spike train synchronicity, we used Pearson’s correlation coefficient:

$$C=\frac{\sum_{i=1}^{n}x_{i}y_{i}}{\sqrt{\sum_{i=1}^{n}x_{i}^{2}\sum_{i=1}^{n}y_{i}^{2}}}$$ (12)

where *x*_*i*_ and *y*_*i*_ denoted the two zero-mean vectors of two spike trains. For identical vectors *x*_*i*_ and *y*_*i*_, the correlation C was equal to unity. Since we were interested in millisecond synchronization, the bin size was set to 1 ms.

The firing rate of individual neurons was estimated as the average spike count per second. The mean network firing rate was then obtained by averaging the firing rates of all neurons in the network. The values in different groups were compared using the Mann-Whitney U-test and a p < 0.01 was considered statistically significant.

## Results

The striatum is a purely inhibitory recurrent network driven by excitatory inputs from the cortex and thalamus. An important feature of the striatum network is that the FF inhibition from FSIs to MSNs and FB inhibition from MSNs to other MSNs are clearly segregated. Individual striatal neurons do not act independently to affect rhythmic population activity, and here we use a spiking neuronal network model to study how they interact with each other and time-dependent external stimuli to induce oscillatory activity in the MSN population.

### Propagation of cortical oscillations in the striatal network

**Fig 2:**
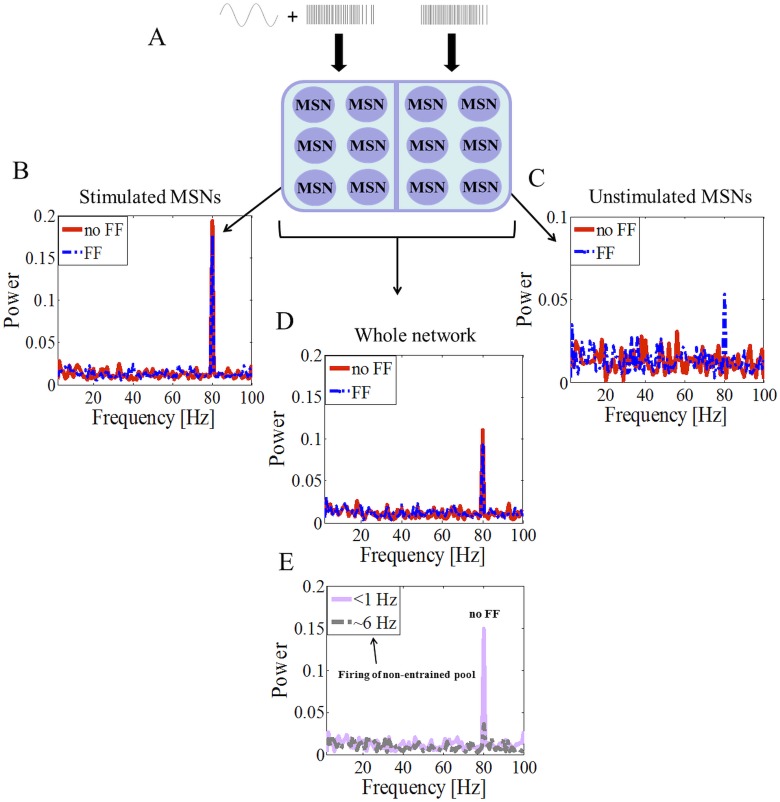
Interplay between oscillatory inputs into MSNs and FSIs. (A) Schematic of striatum network. The stimulated MSNs received sinusoidal current inputs in addition to the Poisson type background spiking input. The unstimulated-MSNs only received the Poisson type spiking inputs. (B) The spectral analysis of the population activity of MSNs, without (red trace) and with (blue trace) feedforward inhibition, stimulated by a sinusoidal currents input at 80 Hz. (C) Same as in B but for unstimulated MSNs. (D) The spectral analysis of the whole MSN population. (E) In the absence of FSI stimulation, oscillations in the whole MSN network could be destabilized by the increase in the average firing of unstimulated MSNs.

When a fraction of MSNs was stimulated with sinusoidal inputs at 80 Hz in the addition to the background input, while the rest of MSNs received only the Poisson inputs (Fig 2A), the averaged activity of the whole MSN pool followed the sinusoidal input (Fig 2D, red line). A detailed analysis however revealed that the input driven oscillatory activity was limited only to the directly stimulated MSNs (Fig 2B, red line) and unstimulated neurons did not show any oscillatory activity (Fig 2C, red line). That is, partial stimulation of MSNs population was not sufficient to distribute the oscillatory input to the unstimulated MSNs. Moreover, the entrainment of the stimulated MSNs population was affected by the baseline activity of the unstimulated neurons. High firing rate (~6Hz) of the unstimulated MSN population reduced the firing rate of the stimulated MSNs and impaired the transmission of cortical oscillations even to the directly stimulated MSNs (Fig 2E). High firing rate and asynchronous activity of unstimulated MSNs increased the noise in the stimulated MSNs and reduced the signal-to-noise ratio (SNR) of the cortical drive to the stimulated neurons. The oscillations transmission could be restored by matching the firing of the stimulated population with the firing of the unstimulated MSN population. Crucially, in neither of these scenarios, the stimulated MSN population was able to transfer oscillations to the unstimulated population. By contrast, when FSIs (50%) were also stimulated with the same oscillatory inputs as the stimulated MSNs, oscillations were observed not only in the FSIs but also in the MSNs (0.015 vs 0.053, p<0.01, Fig 2C, blue traces). These results showed that FSIs play an important role in distributing cortical input to the striatal MSNs.

**Fig 3:**
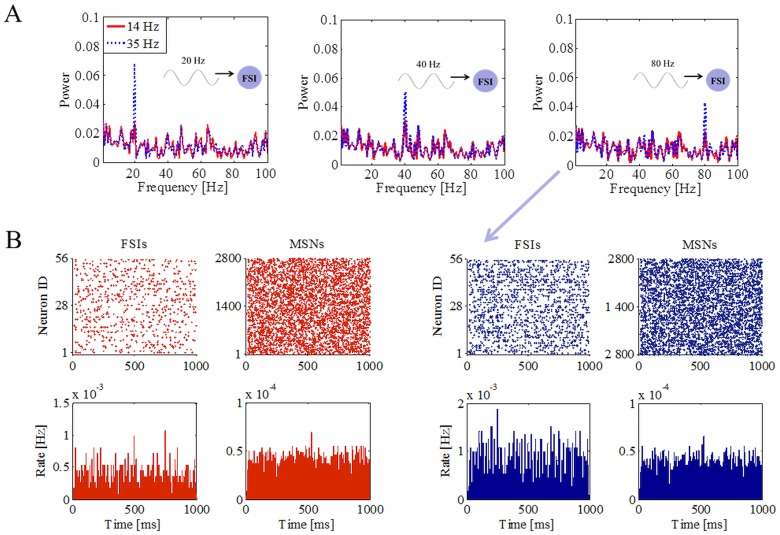
Propagation of asynchronous cortical oscillations in a striatal network model through FSIs. (A) Power spectra of the MSNs population when only FSIs were driven by cortical oscillatory inputs at 20, 40 and 80 Hz. The ability of FSIs to transfer oscillations to MSNs (blue traces) directly depended on their average firing rates. (B) Spiking activity (top) and population firing rate (bottom) of 56 FSIs and 2800 MSNs for the low (A_max_ = 150 pA) and high (A_max_ = 300 pA) maximal oscillatory amplitude onto FSIs. Population firing rate was also divided by the number of corresponding FSIs and MSNs, respectively, and the number of bins.

Next we tested whether stimulation of FSIs alone was sufficient to transmit the cortical oscillations to MSNs over several frequencies (Fig 3). We found that when only FSIs were stimulated, the spectral peak in the MSN population activity directly depended on the average firing of FSIs and the frequency of input oscillations. For instance, FSIs average firing frequency higher than 14.5 ± 3.3 Hz was necessary to transmit 20 Hz cortical oscillations to unstimulated MSNs (Fig 3A). Similar results were obtained for 40 Hz and 80 Hz input frequencies in the case of low (red curve) and high (blue curve) firing rates of FSIs. Previous experimental work [17, 39, 57] has demonstrated the importance of 80 Hz corticostriatal oscillations for shaping neuronal interactions between cortex and striatum. Recently, it has been found that the FSIs fire in relation to very high cortical frequencies [43]. Given the functional relevance of high frequency oscillation in the striatum, in the following we specifically focus on the transmission of 80 Hz oscillation from the cortex to MSNs.

### Firing of FSIs controls propagations of cortical oscillations in the striatal network

Because connection between MSNs and FSIs are only unilateral and FSIs also receive incoherent oscillatory inputs, they exhibit asynchronous activity irrespective of the input amplitude (Fig 3B). Raster plot and firing rate histogram of MSNs, when FSIs fired at low (Fig 3B, left red) or high (Fig 3B, right blue) frequencies, did not show any clear temporal pattern. In these two examples even though the firing rate of FSIs was doubled the average firing of MSNs changed only by a small amount (1.62 ± 1.31 Hz versus 1.53 ± 1.26 Hz). Given this, how are FSIs able to facilitate the transmission of cortical oscillations?

**Fig 4:**
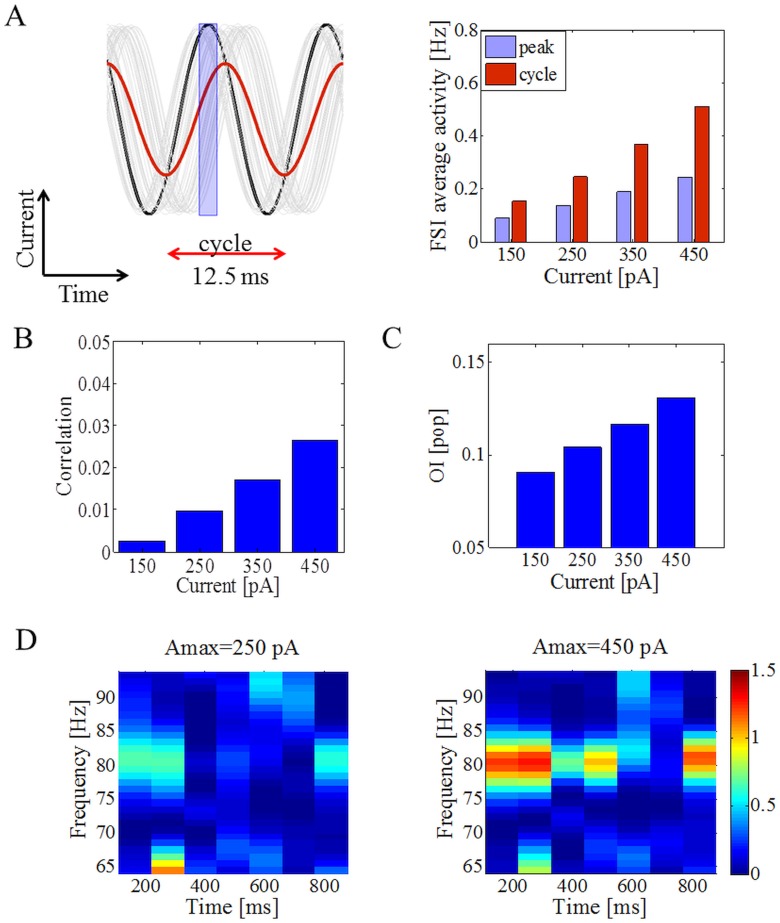
Firing of FSIs and its influence on the transfer of oscillations in the MSN population. (A) The relationship between oscillatory inputs (80 Hz) to FSIs for one oscillatory cycle that spans over 12.5 ms. The thin gray lines and the black line (as a particular example) show the oscillatory inputs to individual FSIs for one trial and red line corresponds to the averaged oscillatory inputs (averaged gray lines) to the FSIs. Average firing of FSIs over the whole oscillation cycle (red bars) as a function of the amplitude of the oscillatory inputs (right panel). The proportion of neurons that spiked in relation to the oscillation peaks (defined in the range over ±2 ms of the peak value during each cycle; marked by the purple rectangle) also increased with an increase in the oscillatory amplitude. (B) The average pairwise correlation in the FSI population increased as a function of sinusoidal current amplitude. (C) Oscillatory index of the MSN population increased as a function of the sinusoidal current amplitude. (D) Temporal fluctuations in the transfer of oscillations from FSIs to MSNs. In the presence of the weak sinusoidal drive to FSIs, oscillation transfer was not reliable over time as indicated by fluctuations in the amplitude of the spectral peak as a function of time. The stability and strength of oscillations increased for higher values of A_max_. Colors in the spectrograms indicate the power of oscillations. The MSNs were driven by excitatory and uncorrelated Poisson synaptic inputs.

A detailed analysis of the FSIs activity showed that only a few FSIs spiked relative to the oscillation cycle (Fig 4A) when oscillations were successfully transferred to the MSNs. Each FSI had its own preferred firing time relative to other FSIs. The proportion of FSIs (Fig 4A, right panel, purple bars) that fired in relation to the individual peaks of each FSI separately (defined in a range of over ±2 ms of the maximal value during each cycle; Fig 4A, left panel, purple rectangle) also increased with an increase in the maximal input oscillatory amplitude. Together with the firing rate changes, increasing the strength of oscillatory inputs also increased pairwise correlations among FSIs (Fig 4B). Even though the correlation magnitude was small, it was sufficient to influence the firing pattern of MSNs because of the high divergence of FSI inputs to the MSNs. The changes in the firing pattern of individual FSIs with respect to the oscillation phase (they preferentially discharged at the descending phase of the cortical oscillations) and correlations resulted in imprinting the FSIs oscillatory activity pattern on to the MSNs. Consistent with this, the OI of the MSN population showed a steady increase correlated with the higher values of the maximal oscillatory input current (for A_max_ = 150 pA, the peak was not visible, while for A_max_ = 250 pA, it was already quite prominent) (Fig 4C). When FSIs were driven with a weak input it is not that the oscillations were never transferred to the MSNs. A spectrogram of the MSN population activity revealed that when FSIs spiked at low firing rates, given the noise due to Poisson inputs and recurrent connectivity of MSNs, the transmission of oscillations was not stable and oscillations were observed intermittently. That temporal instability of the oscillations was the reason for the weak power of oscillations in MSNs and smaller OI when FSIs spiked at low rates. As the firing rate of the FSIs increased, the transmission became more stable as it became stronger than the noise, and therefore resulting in higher oscillations power and OI (Fig 4D). Finally, when FSIs were driven with synchronous oscillatory inputs the peak at the driving frequency significantly increased (0.0357 vs 0.0497, p<0.001; A_max_ = 250). Increase of the inhibitory rise constant had a similar effect and augmented the peak of the oscillations in the MSN population.

### Effect of feedforward inhibition on the transfer of oscillations to the MSN population

**Fig 5:**
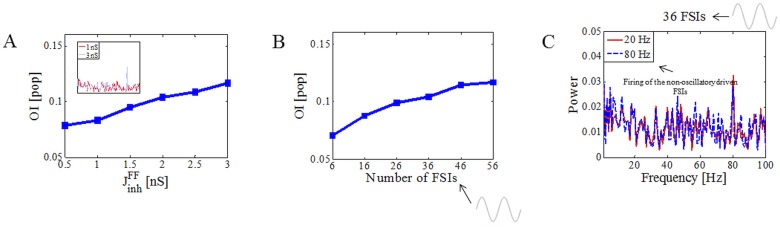
Sensitivity of the oscillations in the MSN population to the parameters of the FF inhibition. (A) Increase in the strength of FF inhibition enhanced the transfer of oscillations from FSIs to MSNs as indicated by a monotonic increase in the OI of MSNs. (B) OI of MSNs also monotonically increased as a fraction of stimulated FSIs increased. (C) Influence of the background activity in unstimulated FSIs (those that did not receive any sinusoidal inputs) on the transfer of oscillations to the MSN population. Maximal amplitude of the oscillatory inputs was equal to 350 pA (mean ± std firing of FSIs was equal to 30.98 ± 4.2 Hz) and was kept constant throughout all simulations.

In our model, cortical oscillations are first transferred to the FSI activity and then imprinted on to the activity of MSNs. That is, once oscillations are established in FSIs, e.g. by increasing the drive to FSIs, the transmission of the oscillations depends on the effective FSIs inputs to the MSNs. The effective FSIs input to MSNs in turns depends on the number of FSIs inputs to individual MSNs, strength of FF inhibition (J^FF^_inh_), and area of the IPSP. Indeed, increasing the strength of the FF inhibition (J^FF^_inh_) increased the OI (Fig 5A). A similar effect would be observed if we changed the connection probability between FSIs and MSNs (data not shown). For the specific choice of J^FF^_inh_, connection probability and cortical input drive (A_max_) within a physiologic range, oscillations could be transferred to MSNs when as little as 16 FSIs (28% of the local FSI population) were driven by cortical inputs (Fig 5B). The maximum amplitude of the oscillatory inputs (A_max_) was set to 350 pA (mean ± std firing of FSIs was equal to 30.98 ± 4.2 Hz) and was kept constant throughout all simulations. When only a fraction of FSIs were stimulated the irregular spiking activity of unstimulated FSIs could slightly impair the transmission of oscillations by adding noise to the oscillatory FSIs inputs to MSNs. Fig 5C shows that when 36 FSIs received oscillatory input on the top of the Poisson background activity (see Materials and methods), and the rest of the FSIs received only Poisson drive that made them fire at two different frequencies, ~20 Hz and ~80 Hz, respectively. In the latter case, the peak was smaller but not significantly (0.033 vs 0.028, p = 0.017).

### Interplay between the sole MSN network parameters and asynchronous oscillatory drive

**Fig 6:**
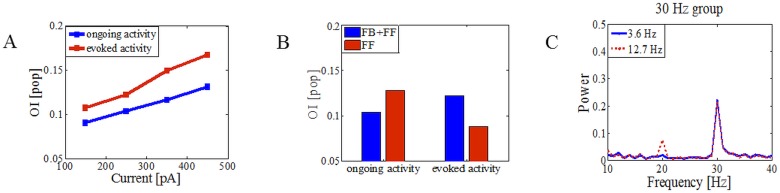
Sensitivity of the oscillations in the MSN population to the parameters of the FB inhibition. (A) OI of MSNs for different values of the maximal oscillatory amplitude onto FSIs when MSNs underwent ongoing (1.56 Hz) or evoked (8.59 Hz) activity. (B) Influence of the FB inhibition on the transfer of the oscillations in the MSN population (A_max_ = 250 pA). (C) The MSN population was split into two equal sized populations. All neurons received Poisson background inputs and, additionally, the first population (referred to as the source network) received asynchronous oscillatory inputs with the driving frequency set at 20 Hz, and the other population (referred to as the target network) also received asynchronous oscillatory inputs but with the driving frequency set at 30 Hz. For the high activity state of the source network, a statistically significant peak was present in the power spectrum of the target network at the oscillatory frequency of the source network (0.0192 vs 0.0752, p<0.01).

The oscillations when transferred with the aid of FSIs are robust to the firing rate of the MSNs. In fact, higher firing rate of MSNs (evoked activity) increased the power of oscillations compared to the low firing rate (ongoing activity) (Fig 6A). This suggests that increase in the firing rate of MSNs during a behavioral task will improve the transfer of cortical oscillations. When we completely removed FB inhibition, the change of the oscillatory peak in the power spectrum depended on the firing regime of the MSNs (Fig 6B). When MSNs fired with low frequency (< 2Hz) the peak increased as the firing of MSNs increased after removing FB inhibition (1.56 Hz vs 1.68 Hz). In contrast, when higher neural activity was imposed (>10 Hz) the peak decreased after removing FB inhibition due to additional desynchronization of MSNs that overrode the effect of increase in the firing rate. Finally, we wanted to see how selective stimulation of only MSNs with oscillatory currents lead to the transfer of oscillations. First, the stimulation was partial and only a portion of MSNs received oscillatory (A_max_ = 250pA) and Poisson-type synaptic background inputs while the rest of the MSN network received only Poisson-type synaptic background inputs and fired less than 1 Hz on average (see Materials and methods). For 6.25% of the oscillatory driven MSN population, the peak at driving frequency was not visible, while for 12.5% it was strong and stable (but could not propagate oscillations into unstimulated MSN pool even when oscillatory driven MSNs fired very high). Further we split MSNs into two populations. All neurons received Poisson background inputs and, additionally, the first half of MSNs received oscillatory inputs with the driving frequency set to 20 Hz, and the other half also received oscillatory inputs but with the driving frequency set to 30 Hz (in order to avoid interference of the first and second harmonics). We referred to the first half of the MSN population as the source network and the second half as the target network [58]. Here, we investigated how the source network affected the oscillatory activity in the target network. Fig 6C shows that the activity of the source network can impose the oscillatory activity in the target network, depending on the firing profile of the source network. When the average firing in the source network was ~ 3.6 Hz, the source network could not impose oscillatory activity at its driving frequency onto the target network (Fig 6C, blue line). We found that when the source network was in a state of high activity, a statistically significant peak was present in the power spectrum of the target network at the oscillatory frequency of the source network (Fig 6C, red line; 0.0192 vs 0.0752, p<0.01).

## Discussion

Oscillations are a ubiquitous feature of the population activity in the brain. Signatures of behavioral tasks and brain diseases (such as Parkinson's disease) can be observed in the dynamics of oscillatory activity of the BG [37]. Therefore, there is a great interest in understanding the origin and modulation of oscillatory activity in the BG. While the mechanisms underlying the oscillatory activity of the subthalamic nucleus—globus pallidus (external segment) network are relatively well understood [59–61], the origin of oscillations in the striatum is poorly understood. Purely inhibitory networks such as the striatum can only generate oscillations when recurrent connectivity is dense and strong [40]. Therefore, it is likely that striatal oscillations are either generated by sub-threshold resonance of the striatal neurons or imposed by the cortico-thalamic projections. Here, we explored the later possibility and show that fast spiking interneurons could play a major role in transferring cortical oscillations to the striatal projection neurons.

We have shown that the selective activation of striatal FSIs via asynchronous oscillatory inputs could successfully entrain the MSN population to oscillate at the input driving frequency. Strong and divergent connectivity of FSIs implies that even weak oscillations in FSI population activity can be spread to the whole MSN population.

Here we have used a reduced model of the striatum and simplified neuron models, yet the model is powerful enough to include the effects of neuro-modulators. In general, neuro-modulators such as acetylcholine (Ach) and dopamine affect the network activity by modulating the effective strength of cortical excitation, feedback and feedforward inhibitions [53]. In our model by changing the strength of the feedforward and feedback inhibition we can speculate on how ACh and dopamine may affect the transfer of oscillations. For instance, ACh while weakens the strengths of FSIs synapses onto the MSNs, it depolarizes the FSIs and increases their firing rate [62]. Reduction in the synaptic strength and increased firing rate will increase the mean and reduce the variance of the FF inhibition. That is, increase in the firing rate of the cholinergic neurons should increase the transfer of cortical oscillation via FSIs. Similarly, in PD dopamine depletion increases the connectivity between FSIs and D2 type dopamine expressing MSNs [54]. Such a change is proposed to be a compensatory mechanism to reduce the firing rate of the D2-MSNs [45]. However, our model here suggests that such an increase in the connectivity between FSIs and D2 MSNs would also increase the spread of cortical oscillations in the MSN population. That is, our model predicts that in dopamine depleted state there should be higher correlation between the cortical and striatal oscillations [17].

FSIs and feedforward inhibition are a common property of neuronal networks throughout the brain and play crucial role in neural computations. For instance, FF inhibition sets the window of temporal integration and spiking and thereby contributes to the control of firing rate and correlations. Moreover, FSIs provides a simple circuit mechanism to gate the propagation of spiking activity [63–64]. Furthermore, by setting a narrow window of spiking FSIs can synchronize the neuronal activity and generate high frequency cortical rhythms [65–67]. Finally, by directly projecting to the soma of the neurons, FSIs control the effective gain of the neurons and provide the much needed divisive inhibition and a mechanism of normalization [68–70]. In the striatum despite their high firing rates, FSIs do not seem to play a major role in controlling the firing of MSNs [35] and so far it has not been possible to attribute a functional role to FSIs in the striatum. Here, we have proposed that FSIs can perform an important role in transferring cortical oscillations to the striatum especially to those MSNs that are not directly driven by the cortical oscillations. Further, we have identified multiple factors such as the level of feedback and feedforward inhibition that influence the transfer of oscillations to MSNs. Especially, we have quantified the effect of number of activated neurons (MSNs, or FSIs), ongoing activity, connectivity, and synchronicity of inputs. Our results support the idea of the precise orchestration of FSI activity that plays a key role in determining the pattern of the firing of MSNs, which might provide optimal integration of external inputs into striatal network.
